# Calcium trafficking and gastrointestinal physiology following an acute lipopolysaccharide challenge in pigs

**DOI:** 10.1093/jas/skae073

**Published:** 2024-03-14

**Authors:** Julie Opgenorth, Edith J Mayorga, Megan A Abeyta, Brady M Goetz, Sonia Rodriguez-Jimenez, Alyssa D Freestone, Chad H Stahl, Lance H Baumgard

**Affiliations:** Department of Animal Science, Iowa State University, Ames, IA 50011, USA; Department of Animal Science, Iowa State University, Ames, IA 50011, USA; Department of Animal Science, Iowa State University, Ames, IA 50011, USA; Department of Animal Science, Iowa State University, Ames, IA 50011, USA; Department of Animal Science, Iowa State University, Ames, IA 50011, USA; Department of Animal Science, Iowa State University, Ames, IA 50011, USA; Department of Animal and Avian Sciences, University of Maryland, College Park, MD 20742, USA; Department of Animal Science, Iowa State University, Ames, IA 50011, USA

**Keywords:** endotoxemia, gut motility, inflammation, hypocalcemia

## Abstract

The influence of systemic immune activation on whole-body calcium (**Ca**) trafficking and gastrointestinal tract (**GIT**) physiology is not clear. Thus, the study objectives were to characterize the effects of lipopolysaccharide (**LPS**) on Ca pools and GIT dynamics to increase understanding of immune-induced hypocalcemia, ileus, and stomach hemorrhaging. Twelve crossbred pigs [44 ± 3 kg body weight (**BW**)] were randomly assigned to 1 of 2 intramuscular treatments: (1) control (**CON**; 2 mL saline; *n* = 6) or (2) LPS (40 µg LPS/kg BW; *n* = 6). Pigs were housed in metabolism stalls to collect total urine and feces for 6 h after treatment administration, at which point they were euthanized, and various tissues, organs, fluids, and digesta were weighed, and analyzed for Ca content. Data were analyzed with the MIXED procedure in SAS 9.4. Rectal temperature and respiration rate increased in LPS relative to CON pigs (1.4 °C and 32%, respectively; *P* ≤ 0.05). Inflammatory biomarkers such as circulating alkaline phosphatase, aspartate aminotransferase, and total bilirubin increased in LPS compared with CON pigs whereas albumin decreased (*P* ≤ 0.02). Plasma glucose and urea nitrogen decreased and increased, respectively, after LPS (43% and 80%, respectively; *P* < 0.01). Pigs administered LPS had reduced circulating ionized calcium (**iCa**) compared to CON (15%; *P* < 0.01). Considering estimations of total blood volume, LPS caused an iCa deficit of 23 mg relative to CON (*P* < 0.01). Adipose tissue and urine from LPS pigs had reduced Ca compared to CON (39% and 77%, respectively; *P* ≤ 0.05). There did not appear to be increased Ca efflux into GIT contents and no detectable increases in other organ or tissue Ca concentrations were identified. Thus, while LPS caused hypocalcemia, we were unable to determine where circulating Ca was trafficked. LPS administration markedly altered GIT dynamics including stomach hemorrhaging, diarrhea (increased fecal output and moisture), and reduced small intestine and fecal pH (*P* ≤ 0.06). Taken together, changes in GIT physiology suggested dyshomeostasis and alimentary pathology. Future research is required to fully elucidate the etiology of immune activation-induced hypocalcemia and GIT pathophysiology.

## Teaser

Immune activation causes hypocalcemia and inappetence. Thus, this study aimed to enhance our understanding of calcium (**Ca**) trafficking and gastrointestinal tract dynamics after lipopolysaccharide (**LPS**) administration. Results indicate that LPS caused stomach hemorrhage, increased large intestine motility, and marked hypocalcemia, but it is not clear where circulating Ca is sequestered.

## Introduction

Lipopolysaccharide (**LPS**), or endotoxin, is a cell wall component in Gram-negative bacteria that elicits an inflammatory response and can lead to organ damage and shock (Danner et al., 1999). A well-characterized species conserved response to endotoxemia is hypocalcemia (Carlstedt et al., 2000; Toribio et al., 2005). The circulating pool of ionized calcium (**iCa**) decreases and reaches a nadir within hours of an LPS insult (Carlstedt et al., 2000; Horst et al., 2020). Upon activation, leukocytes, platelets, and endothelial cells absorb extracellular calcium (**Ca**) to exert effector functions (Varga-Szabo et al., 2009). However, Ca uptake into activated cells is unlikely to account for the magnitude of hypocalcemia (Carlstedt et al., 2000; Waldron et al., 2003; Straub, 2015).

Better characterizing the etiology behind endotoxin- induced hypocalcemia may have practical implications to our understanding of how (and why) mineral metabolism is altered by infection. Some studies report increased Ca concentrations in ascites fluid (Carlstedt et al., 2000; He et al., 2020), liver (He et al., 2020), and muscle (Benson et al., 1989; Bhattacharyya et al., 1993) following immune activation. Further, we and others have described how systemic immune activation can increase intestinal permeability (Guo et al., 2013; Mayorga et al., 2020) and negatively influence appetite and gastrointestinal tract (**GIT**) motility (De Winter and De Man, 2010; Mani et al., 2012). Thus, our objectives were 2-fold: the first was to quantify Ca pools in tissues, organs, fluids, and GIT lumen contents to characterize Ca partitioning and sequestration during immune activation. A secondary aim was to evaluate how immune activation broadly alters GIT physiology.

## Materials and Methods

### Experimental design

All procedures were approved by the Iowa State University Animal Care and Use Committee. Twelve crossbred barrows [44 ± 3 kg body weight (**BW**)] were randomly assigned to 1 of 2 treatments: control (**CON**; 2 mL sterile saline; *n* = 6) or LPS (40 µg/kg BW in 2 mL saline; *Escherichia coli* O55:B5; Sigma-Aldrich Corp., St. Louis, MO; *n* = 6). Pigs were housed in individual metabolism stalls at the Iowa State University Swine Nutrition Research Farm (Ames, IA) and given 7 d to acclimate where feed intake, rectal temperature, and respiration rate were recorded daily. All pigs were fed ad libitum with a diet formulated to meet or exceed all nutrient requirements established by the NRC (NRC, 2012; Table 1). Following acclimation, feed and water were removed for 2 h before treatment administration and remained withdrawn during the 6-h collection period. Baseline BW and blood samples were obtained prior to treatment administration. LPS was dissolved in sterile saline and filtered through a 0.2-µm sterile syringe (Thermo Scientific, Waltham, MA) and the intramuscular LPS dose was selected based upon previous reports (Leininger et al., 2000; Frank et al., 2005). Both CON and LPS treatments were administered into the gluteal muscle. Following treatment administration, pigs were placed in clean metabolism stalls equipped with urine and feces collection capabilities. Rectal temperature and respiration rate were recorded hourly. At the end of the 6-h challenge, pigs were weighed, blood sampled, and immediately euthanized with captive bolt before exsanguination.

**Table 1.** Ingredient composition of diet fed during acclimation

Item	%
Ingredient composition	
Corn	72.78
Soybean meal (47.5% CP)	23.20
Soybean oil	0.80
Limestone	0.92
Monocalcium phosphate	1.12
NaCl	0.40
Lysine HCl	0.30
Methionine	0.06
Threonine	0.07
Vitamin Premix^1^	0.20
Mineral premix^2^	0.15

^1^Vitamin premix provided the following per kilogram of complete diet: 6,125 IU vitamin A, 700 IU vitamin D3, 50 IU vitamin E, 3 mg vitamin K, 11 mg riboflavin, 56 mg niacin, 27 mg pantothenic acid, 24 mg vitamin B12.

^2^Mineral premix provided the following per kilogram of complete diet: 165 mg Fe (ferrous sulfate), 165 mg Zn (zinc sulfate), 39 mg Mn (manganese sulfate), 16.5 mg Cu (copper sulfate), 0.3 mg I (calcium iodate), 0.3 mg Se (sodium selenite).

### Blood sample collection and analysis

Blood samples obtained at 0 and 6 h relative to treatment administration were collected via jugular venipuncture into disposable tubes (BD vacutainer; Franklin Lakes, NJ). Blood samples collected in tubes containing heparin (BD vacutainer) were immediately analyzed with an iSTAT handheld analyzer and cartridge for iCa (CG8+, Abbott Point of Care, Princeton, NJ). Blood samples collected in tubes with a serum clot activator (BD vacutainer) were allowed to clot for 1 h and were placed in a centrifuge at 1,500 × *g* for 15 min at 4 °C to harvest serum prior to storing at −20 °C until analysis. Serum samples were analyzed by the Iowa State University Veterinary College Clinical Pathology laboratory for concentrations of Na, K, Cl, Ca, P, Mg, Na, HCO_3_, blood urea nitrogen (**BUN**), creatinine, glucose, total protein, albumin, aspartate aminotransferase (**AST**), alkaline phosphatase, gamma-glutamyl transferase (**GGT**), and total bilirubin.

### Tissue collection and analysis

Following euthanasia, bile, ascites fluid, and urine within the bladder were promptly collected with syringes. Muscle and adipose samples were collected from the longissimus muscle and subcutaneous adipose from the dorsal region of the neck, respectively. The liver, heart, spleen, pancreas, and kidneys were collected and weighed as whole tissues and were homogenized prior to sample analysis. Gathering digesta from each segment of the GIT involved removing the stomach by clamping at the esophageal and pyloric sphincters, the small intestine by clamping at the pyloric sphincter to the ileocecal junction, and the large intestine by clamping the ileocecal junction to approximately 10 cm proximal to the rectum. Total contents from each GIT section, including excreted fecal content during the 6-h collection period, were emptied and weighed. Contents were then homogenized before retaining a subsample for Ca analysis and pH measurement. The total volume of urine excreted in the 6-h collection period was quantified with a graduated cylinder and homogenized to collect a subsample. Due to discrepancies in bladder and excreted urine Ca concentrations, it was presumed that excreted urine contained some Ca contamination from the crate (despite pre-acid washing in an attempt to eliminate this possibility). Thus, Ca concentrations from urine within the bladder were used as a proxy for urine Ca quantification. Organs, tissue, fluid, and digesta Ca were analyzed via inductively coupled plasma mass spectrometry by the Iowa State University Veterinary Diagnostics Laboratory on a wet basis. Samples were additionally analyzed for Na, Mg, P, K, Cr, Mn, Fe, Co, Cu, Zn, Se, Mo, and Cd concentrations, and are reported in Supplementary Tables S1–S13. To improve clarity, Ca concentrations were converted to a dry matter (**DM**) basis for tissues, digesta, and feces. Photographs of each stomach were captured and analyzed with the image histogram in Fiji to quantify stomach discoloration and hemorrhaging (Schindelin et al., 2012). The mode of each intensity histogram served as an indicator of stomach hemorrhage.

### Calculations and statistical analysis

Total Ca content in tissues, organs, fluids, and digesta was estimated by multiplying the analyzed Ca concentration by the total parameter weight. Pancreas, ascites, bile, and bladder urine were excluded from attempting to estimate the total Ca quantity due to our inability or lack of confidence in collecting the total sample. Total skeletal muscle weight was estimated with pig carcass weight (74% of live BW; Boler, 2014), considering 67.1% of carcass weight is skeletal muscle in a 45 kg pig (Lonergan et al., 2019). Similarly, total adipose tissue weight was estimated assuming 9.5% of the carcass was fat (Lonergan et al., 2019). To quantify total circulating iCa, complete blood volume was estimated at 67 mL per kg BW (Hannon et al., 1985). Actual equations to estimate muscle and adipose mass and blood volume are as follows:


$$\begin{array}{l} \text{Muscle}\;\text{mass}=\text{Carcass}\;\text{weight}(74\%\times \text{kg}\;\text{of}\;\text{live}\;\text{BW})\times 67.1\% \end{array}$$

$$\begin{array}{l} Adipose\;mass=Carcass\;weight(74\%\times kg\;of\;live\;BW)\times 9.5\% \end{array}$$

$$\begin{array}{l} Blood\;volume=Total\;blood\;volume(67\;mL\times kg\;of\;live\;BW) \end{array}$$

Data were analyzed with the MIXED procedure in SAS 9.4 (SAS Institute Inc., Cary, NC). Rectal temperature and respiration rate were analyzed with an autoregressive covariance structure with fixed effects of treatment, time, and their interaction and repeated measures of time with pig as subject. Single measures of circulating parameters taken 6 h after treatment administration were analyzed with the fixed effect of treatment. Rectal temperature, respiration rate, and circulating parameter analysis included a covariate measurement taken immediately prior to treatment administration. Ca concentration, total content analyses in tissues, organs, fluids, and digesta, stomach image intensity, and digesta pH did not include a covariate. Results are reported as least squares means and standard error of the mean and considered significant when *P* ≤ 0.05 and a tendency when 0.05 < *P* ≤ 0.10. The sample size was determined by considering logistical constraints and prior research (Horst et al., 2020). Post hoc power analysis (PROC POWER; SAS Institute Inc.) based on a primary parameter of interest (iCa) indicated a statistical power of 98% (α = 0.05).

## Results

**Figure 1.**
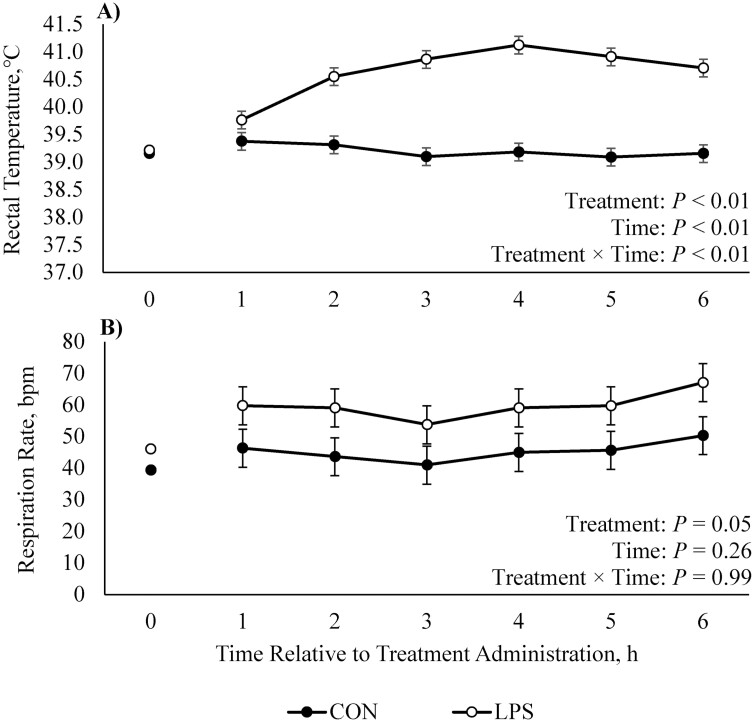
Effects of CON (2 mL saline) or LPS (40 µg/kg) on (A) rectal temperature and (B) respiration rate (breaths per minute; bpm) in growing pigs. Values obtained at 0 h (immediately prior to treatment administration) were utilized as a covariate. Data are represented as least squares means ± standard error of the mean.

Pigs receiving LPS were visibly malaise: four vomited within the first hour and LPS caused increased (22.5%) fecal moisture compared to CON feces (*P* = 0.01; data not shown), indicative of diarrhea. Administering LPS markedly increased rectal temperature relative to CON (1.4 °C; *P* < 0.01) with a peak of 41.1 °C at 4 h (Figure 1A). Additionally, the respiration rate was elevated (32%) throughout the 6-h LPS challenge (*P* = 0.05; Figure 1B).

The effects of LPS on circulating metabolic and inflammatory parameters are reported in Table 2. Albumin and glucose were decreased in LPS compared to CON pigs (8% and 43%, respectively; *P* ≤ 0.02). Pigs administered LPS had increased alkaline phosphatase, BUN, AST, and total bilirubin relative to CON (98%, 80%, 64%, and 183%, respectively; *P* ≤ 0.01). Additionally, GGT, hemoglobin, and hematocrit tended to be increased in LPS compared to CON pigs (25%, 10%, and 9%, respectively; *P* ≤ 0.08). However, no overall treatment difference was detected in creatinine, blood gas, or pH parameters (*P* > 0.17).

**Table 2.** Effects of LPS on blood parameters 6 h after administration

Parameter	Treatment		SEM	*P*
	CON^1^	LPS^2^		
Clinical parameters				
Albumin, g/dL	3.99	3.68	0.08	0.02
Alkaline phosphatase, IU/L	181	358	15	<0.01
AST, IU/L	74	121	9	<0.01
BUN, mg/L	8.3	14.9	0.7	<0.01
Creatinine, mg/dL	1.13	1.81	0.29	0.17
GGT, IU/L	44.3	55.4	3.9	0.08
Glucose, mg/dL	111	63	6	<0.01
Total protein, g/dL	6.12	5.93	0.11	0.24
Total bilirubin, mg/dL	0.53	1.50	0.20	<0.01
Minerals				
iCa, mmol/L	1.28	1.09	0.03	<0.01
Ca, mg/L	9.98	9.22	0.24	0.06
Cl, mEq/L	101	101	1	0.73
K, mEq/L	6.51	6.54	0.34	0.96
Mg, mg/L	2.25	2.40	0.13	0.44
Na, mEq/L	142	142	1	0.84
P, mg/L	11.0	11.3	0.3	0.54
Gases and pH indicators				
sO_2_, %	70.3	73.9	6.7	0.72
pO_2_, mmHg	52.2	50.7	4.7	0.83
pCO_2_, mmHg	51.2	42.7	4.5	0.22
TCO_2_, mmol/L	24.0	22.5	1.7	0.57
Base excess, mmol/L	−4.67	−5.67	2.42	0.78
HCO_3_, mmol/L	22.3	23.2	2.0	0.77
pH	7.26	7.29	0.05	0.61
Hematocrit, %PCV	35.4	38.6	1.2	0.0
Hemoglobin, g/dL	12.0	13.2	0.4	0.07

^1^CON: pigs administered 2 mL sterile saline.

^2^LPS: pigs administered 40 µg/kg BW LPS (*Escherichia coli* O55:B5) in 2 mL saline.

Abbreviations: AST, aspartate aminotransferase; BUN, blood urea nitrogen; GGT, gamma-glutamyl transferase; iCa, ionized Ca; sO_2_%, oxygen saturation; pO_2_, partial pressure of oxygen; pCO_2_, partial pressure of carbon dioxide; TCO_2_, total carbon dioxide.

As expected, LPS reduced circulating iCa (15%; *P* < 0.01; Table 2) and tended to decrease total serum Ca (8%; *P* = 0.06; Table 3). Concentrations of Ca from tissues, organs, digesta, and fluids are provided in Table 3. Ca concentrations were decreased in adipose tissue and urine of LPS compared with CON pigs (39% and 74%, respectively; *P* ≤ 0.04). Total Ca concentrations did not increase after LPS in any tissues, organs, fluids, or digesta. When estimating total Ca content across the pig (Table 4), circulating iCa was decreased by 23 mg in LPS-administered pigs. Total adipose and urine from LPS pigs contained less Ca than CON pigs (39% and 77%, respectively; *P* ≤ 0.05). Despite no difference in kidney Ca concentrations between CON and LPS, the total amount of kidney Ca tended to increase in LPS compared to CON pigs (35%; *P* = 0.08) because kidney weights were larger (31%; *P* = 0.02; Table 5) in LPS pigs. Fecal Ca was substantially increased in LPS relative to CON pigs (2.8-fold; *P* < 0.01; Table 4), ostensibly due to increased fecal output since the total amount of Ca across digesta did not differ by treatment (*P* > 0.74; Table 4). No other changes in total Ca were detected from the analyzed pools post-LPS. While the liver from LPS pigs weighed more (15%; *P* = 0.03; Table 5), total liver Ca did not differ by treatment. Including alterations in total blood, adipose, urine, and kidney Ca, a sum of 110 mg Ca was unaccounted for in LPS-administered pigs.

**Table 3.** Effects of LPS on Ca concentrations 6 h after administration

Location	Treatment		SEM	*P*
	CON^1^	LPS^2^		
Tissues, mg/kg DM				
Adipose	105	64	11	0.03
Heart	408	333	41	0.22
Kidney	416	482	33	0.19
Liver	498	399	40	0.11
Muscle	314	355	28	0.33
Pancreas	710	720	45	0.88
Spleen	333	308	25	0.50
Digesta, g/kg DM				
Stomach Contents	2.12	2.77	0.32	0.18
Small Intestine Contents	2.34	2.04	0.25	0.41
Large Intestine Contents	19.9	26.0	4.8	0.40
Feces	25.8	24.6	1.3	0.55
Fluids, mg/L wet				
Blood (iCa)	51.1	43.5	1.2	<0.01
Ascites	74.1	77.1	3.1	0.51
Bile	209	279	34	0.17
Urine	16.4	4.2	3.7	0.04

^1^CON: pigs administered 2 mL sterile saline.

^2^LPS: pigs administered 40 µg/kg BW LPS (*Escherichia coli* O55:B5) in 2 mL saline.

Abbreviations: DM, dry matter; iCa, ionized Ca.

**Table 4.** Effects of LPS on estimated total Ca pools 6 h after administration

Location	Treatment		SEM	*P*
	CON^1^	LPS^2^		
Tissues, mg				
Adipose	229	139	22	0.02
Heart	17.5	14.2	2.2	0.31
Kidney	14.5	19.6	1.9	0.08
Liver	117	107	10	0.51
Muscle	1,610	1,809	163	0.41
Spleen	6.11	6.33	0.57	0.79
Digesta, mg				
Stomach contents	84	107	37	0.68
Small intestine contents	73.6	62.9	15.7	0.64
Large intestine contents	3,542	2,161	744	0.22
Feces	795	2,237	296	<0.01
Digesta sum	4,382	4,790	833	0.74
Fluids, mg				
Blood (iCa)	146	123	3	<0.01
Urine	2.60	0.60	0.63	0.05

^1^CON: pigs administered 2 mL sterile saline.

^2^LPS: pigs administered 40 µg/kg BW LPS (*Escherichia coli* O55:B5) in 2 mL saline.

Abbreviation: iCa: ionized Ca.

**Table 5.** Effects of LPS on organ weights and urine volume 6 h after administration

Parameter	Treatment		SEM	*P*
	CON^1^	LPS^2^		
Weight				
Heart, g				
Wet	210	203	7	0.53
DM	42.1	42.8	1.5	0.75
Kidney, g				
Wet	172	226	13	0.02
DM	34.8	40.5	1.8	0.05
Liver, g				
Wet	915	1,052	39	0.03
DM	236	272	13	0.07
Spleen, g				
Wet	87.3	98.7	7.5	0.31
DM	18.5	20.8	1.5	0.30
Volume				
Urine, mL	149	213	38	0.26

^1^CON: pigs administered 2 mL sterile saline.

^2^LPS: pigs administered 40 µg/kg BW LPS (*Escherichia coli* O55:B5) in 2 mL saline.

Abbreviation: DM, dry matter.

**Figure 2.**
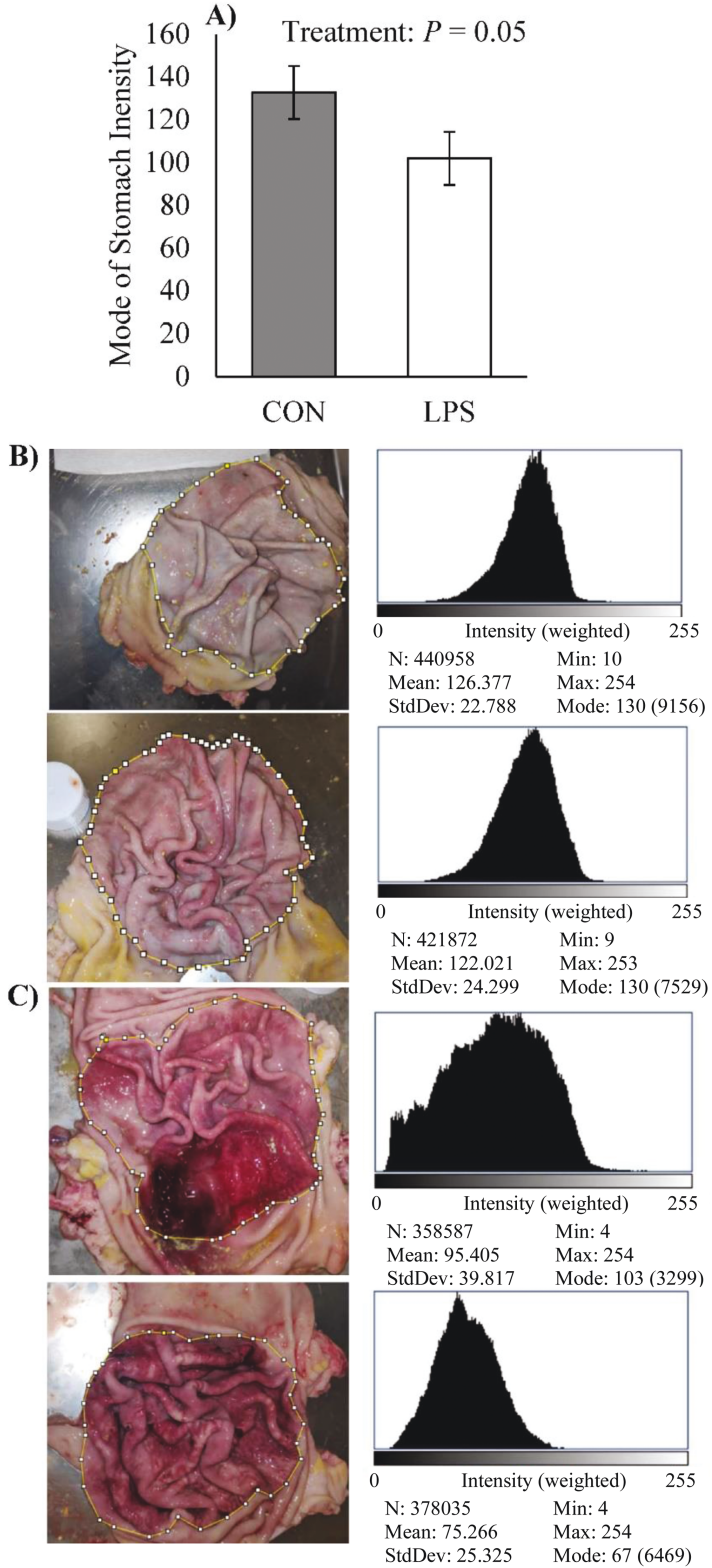
Effects of CON (2 mL saline) or LPS (40 µg/kg) on (A) stomach image intensity from analyzed images of the stomach mucosa in ImageJ Fiji. Data are represented as least squares means ± standard error of the mean. Representative images and intensity histograms of stomachs are displayed from two CON (B) and two LPS (C) pigs.

The mode of stomach image intensity from LPS pigs was decreased (23%; *P* = 0.05; Figure 2A) and indicated LPS caused a darker stomach mucosa (representative images from CON and LPS are reported in Figure 2B and C, respectively). Parameters of GIT physiology are reported in Table 6. The wet and DM weight of gastric and small intestine digesta did not differ by treatment (*P* > 0.28), but large intestine contents were reduced in LPS relative to CON pigs (46% and 49% on wet and DM basis, respectively; *P* ≤ 0.05). Total feces collected during the 6-h LPS challenge was increased in LPS compared with CON pigs (5-fold and 3-fold on wet and DM basis, respectively; *P* = 0.01). The sum amount of digesta contents (GIT segments and feces combined) did not differ by treatment. As a percentage of total collected digesta, LPS large intestine contents were decreased (38% and 39% on wet and DM basis, respectively; *P* ≤ 0.07), and feces were increased (4-fold on wet and DM basis; *P* = 0.02) relative to CON pigs. Small intestine pH tended to decrease in LPS pigs relative to CON (5.87 and 6.43, respectively; *P* = 0.06), and fecal pH was reduced (6.08 and 6.31 in LPS and CON, respectively; *P* < 0.01). Stomach and large intestine pH did not differ by treatment (*P* > 0.43).

**Table 6.** Effects of LPS on GIT physiology 6 h after administration

Parameter	Treatment		SEM	*P*
	CON^1^	LPS^2^		
Weight, g				
Stomach lumen contents				
Wet	197	291	58	0.28
DM	41.4	42.2	16.1	0.97
Small intestine lumen contents				
Wet	282	276	55	0.95
DM	31.8	30.0	6.8	0.85
Large intestine lumen contents				
Wet	874	472	127	0.05
DM	164.7	84.8	23.0	0.04
Feces				
Wet	82	424	80	0.01
DM	31.1	95.8	14.7	0.01
Digesta sum				
Wet	1,435	1,405	155	0.89
DM	266	241	23	0.45
Proportion of GIT contents, %				
Stomach lumen contents				
Wet	15.3	18.2	5.1	0.70
DM	17.7	10.8	5.9	0.43
Small intestine lumen contents				
Wet	19.4	18.8	2.8	0.88
DM	11.9	12.3	2.9	0.92
Large intestine lumen contents				
Wet	59.4	37.1	5.9	0.03
DM	61.0	37.0	8.0	0.07
Feces				
Wet	5.9	25.9	5.2	0.02
DM	9.4	39.9	7.4	0.02
GIT content pH				
Stomach	4.32	4.47	0.13	0.43
Small intestine	6.43	5.87	0.19	0.06
Large intestine	5.83	5.82	0.07	0.93
Feces	6.31	6.08	0.05	<0.01

^1^CON: pigs administered 2 mL sterile saline.

^2^LPS: pigs administered 40 µg/kg BW LPS (*Escherichia coli* O55:B5) in 2 mL saline.

Abbreviations: DM, dry matter; GIT, gastrointestinal tract.

## Discussion

### Calcium trafficking

The etiology of hypocalcemia during immune activation is poorly understood. Some speculate that hypocalcemia is an important survival strategy enabling optimal circulating LPS clearance (Munford et al., 1981). Ca ions bind phosphate groups on adjacent LPS monomers, which stabilize LPS aggregates and impede LPS removal (Harm et al., 2021). The mechanism by which circulating iCa decreases under acute immune activation remains unknown since Ca regulatory hormones are unlikely to account for the rapidity of LPS-induced hypocalcemia (Waldron et al., 2003). Further, while activated leukocytes and platelets take up Ca to facilitate effector functions (Varga-Szabo et al., 2009), Ca influx into intracellular spaces is unlikely to account for the Ca deficit (Carlstedt et al., 2000; Straub, 2015). Therefore, it was of interest to understand how LPS-induced hypocalcemia occurs by identifying where Ca is trafficked during immune activation. The first study objective was to characterize Ca concentrations in body compartments and excreta at the expected circulating iCa nadir following LPS administration. We hypothesized that Ca concentrations would increase in the liver, ascites fluid, and skeletal muscle due to previous research in pigs and rodents during endotoxemia (Benson et al., 1989; Bhattacharyya et al., 1993; Carlstedt et al., 2000; He et al., 2020).

Elevated rectal temperatures and respiration rates in LPS-administered pigs agree with the typical clinical signs of LPS-induced immune activation (Johnson and von Borell, 1994; Kvidera et al., 2017). Further, circulating alkaline phosphatase, AST, GGT, and total bilirubin increased or tended to increase after LPS administration, which is likely due to the liver’s response to LPS detoxification and inflammatory injury (Yu et al., 2012). Serum albumin (a negative acute phase protein [**APP**]) was reduced by LPS administration, and this agrees with an increased acute phase response (Fleck, 1989). Hypoglycemia (a hallmark of endotoxemia across species; Leininger et al., 2000; Horst et al., 2019) developed post-LPS herein; a metabolic consequence of increased glucose uptake by activated immune cells (Lang and Dobrescu, 1991). Additionally, BUN was elevated in LPS pigs, which agrees with other models (Bruins et al., 2003; Kvidera et al., 2017) and indicates increased skeletal muscle amino acid (**AA**) mobilization to supply precursors for gluconeogenesis and APP production (Iseri and Klasing, 2014). The AA profile of muscle differs from APP (Reeds et al., 1994), and the unutilized AA is deaminated and the ammonia enters the urea cycle (Horst et al., 2019). Further, pigs developed diarrhea post-LPS administration, a visual observation confirmed by dehydration (increased hemoglobin and hematocrit concentrations; Billett, 1990) and increased fecal moisture. In summary, pigs administered LPS presented characteristic signs of an intense systemic immune response.

The primary parameter of interest was circulating iCa, which was indeed reduced at 6 h in LPS-treated pigs and the timing and extent agreed with other models (Carlstedt et al., 2000; Toribio et al., 2005; Horst et al., 2020). We hypothesized that Ca would most likely increase in liver and ascites fluid; however, both liver and ascites Ca content were unaltered by LPS. Several stipulations from previous studies may explain why we did not detect increased liver Ca in the current experiment. Carlstedt et al. (2000) primarily observed elevated liver Ca when pigs were parenterally supplemented with Ca during endotoxemia. Further, LPS increased liver Ca after 12 h, but not before, even though circulating Ca was decreased much earlier (Sakaguchi et al., 1984; He et al., 2020). Collectively, this indicates the liver is not a primary location of Ca sequestration during LPS-induced hypocalcemia—at least not acutely.

Fluid accumulation in the peritoneal cavity is often associated with liver injury (Ward, 2016), which is a consequence of endotoxemia (Yan et al., 2014). Ascites Ca increases at 12 h post-cecal ligation (He et al., 2020) and by 6 h post-LPS (Carlstedt et al., 2000). Ascites Ca sequestration is a plausible hypothesis because a proportion of circulating Ca is bound to albumin, which rapidly exits circulation to extravascular spaces as LPS increases vascular permeability (Ince et al., 2016). However, no detectable increase in ascites Ca was detected herein, and anecdotally there was not an increase in total ascites fluid in LPS-administered pigs anyway (visually estimated to be less than 10 mL and not different between treatments). In summary, we did not detect increased hepatic or ascites fluid Ca contents and thus neither of these two pools appear to explain where circulating Ca goes during hypocalcemia. Further, no other analyzed Ca pool appeared to substantially accumulate Ca after LPS.

Total Ca quantified in kidneys tended to increase post-LPS. We and others have observed increased parathyroid hormone (**PTH**) following LPS administration in cows and horses (Toribio et al., 2005; Al-Qaisi et al., 2020). The tendency for elevated kidney Ca in LPS pigs may have resulted from elevated PTH and renal Ca-sensing receptors that prevent urinary Ca excretion by upregulating Ca resorption (Riccardi and Brown, 2010). In support, urinary Ca was markedly reduced in the current experiment, an observation that corroborates others (Toribio et al., 2005; He et al., 2020). Reasons why LPS-infused pigs reduce urinary Ca output are not clear, but obviously, this is not a route that partially explains hypocalcemia and suggests Ca is not excreted but is sequestered during immune activation. Further, LPS can induce kidney inflammation (Han et al., 2012), a likely outcome in the current study since kidney weight was increased by LPS (which ostensibly influenced the total kidney Ca equation). Regardless, the subtle increase detected in kidney Ca (approximately 5 mg) and reduced urine Ca excretion (approximately 2 mg) from LPS pigs is mostly unmeaningful in the grand scheme of the total Ca flux.

Interestingly, LPS decreased adipose tissue Ca concentrations, and this disagrees with prior reports (Carlstedt et al., 2000; Radimerski et al., 2010). Reasons for the inconsistencies among the studies are not clear, but endotoxemia can cause acute adipocyte lipolysis (Wasyluk and Zwolak, 2021), which agrees with increased NEFA post-LPS (Leininger et al., 2000). Since increased adipose Ca appears to downregulate lipolysis (Shi et al., 2001), there could be biological plausibility (to facilitate adipose mobilization) for reducing adipocyte Ca during endotoxemia. Lipolysis post-LPS is mediated in part by increased lipolytic hormones (e.g., cortisol, glucagon, and epinephrine; Bach et al., 2016). Although Ca is an important secondary messenger, it is not required for epinephrine or glucagon signaling (Kuo, 1970). Ca appears to blunt epinephrine’s lipolytic effects through activating phosphodiesterase, which decreases cyclic adenosine monophosphate concentrations and phosphorylation of hormone-sensitive lipase (He et al., 2011). Therefore, decreased adipose Ca may enhance the lipolytic effects of catabolic hormones that increase after LPS administration. Adipose tissue Ca content is also reduced in parallel with circulating Ca in peripartal dairy cows (Horst et al., 1975), a physiological stage accompanied with immune activation and hypocalcemia (Horst et al., 2021). Nevertheless, adipose tissue does not appear to be where Ca is partitioned during immune activation, but understanding how and why adipose tissue Ca decreases following immune has pragmatic implications to periparturient sow and dairy cow nutrition and management decisions.

Muscle Ca was unaltered post-LPS, and this agrees with a previous study in pigs (Carlstedt et al., 2000), but not in rats where immune activation increases muscle Ca influx (Benson et al., 1989; Bhattacharyya et al., 1993). Discrepancies may depend on muscle type analyzed or the large standard error in analysis herein (discussed further below). Increased Ca influx into skeletal muscle is intuitive because Ca enhances proteolysis, which contributes to the AA and energetic needs of immune activation (Benson et al., 1989; Johnson, 2012) and Ca additionally limits muscle glucose uptake as a strategy to encourage “glucose-sparing” for activated leukocytes (Uryash et al., 2022). Further, elevated muscle Ca is a common denominator of myopathies associated with muscle damage or wasting (e.g., sepsis, muscle trauma, and Duchenne muscular dystrophy; Gissel, 2006; Callahan and Supinski, 2009). The large proportion of muscle mass in the carcass makes it a logical sink for sequestering excess Ca from circulation. Although total muscle Ca only numerically increased (12%) in LPS pigs and was far from statistical significance (*P* > 0.4), the standard error of total muscle Ca was larger than the Ca deficit, so our analysis is limited in its ability to precisely detect small but biologically meaningful changes in Ca.

The large intestine contents and feces comprised the largest quantity of Ca, but like muscle, was also accompanied with substantial variability. Large intestine Ca did not differ by treatment, but if the colon takes up ~10% of absorbed dietary Ca (Wasserman, 2004), the potential for CON and LPS pigs to absorb large intestine Ca was 354 and 216 mg (10% of the large intestine Ca content), respectively. This absorptive difference (138 mg) could theoretically contribute to hypocalcemia. Further, feces Ca was increased by LPS, but this is presumably due to increased fecal output since fecal Ca on a concentration basis did not differ by treatment, and the overall sum of Ca from stomach contents to feces was similar by treatment. This suggests an increase in fecal Ca is a consequence of reduced large intestine contents and not necessarily an efflux of circulating Ca. Regardless of the speculation, it further illustrates how difficult it is to accurately characterize Ca flux using a concentration at one moment in time.

Several limitations may have prevented our ability to accurately account for the circulating Ca deficit in LPS pigs: (1) the standard error (likely analytical and not biological) between CON and LPS pigs was in some pools larger than the iCa deficit (i.e., muscle and digesta) that made identifying true statistical differences unfeasible, (2) we analyzed Ca concentrations in a small portion of subcutaneous adipose and skeletal muscle to estimate total Ca pools, which does not account for the diversity in adipose depots and muscle fiber types that may be influenced differently by systemic immune activation, (3) some samples were unable to be fully collected and thus quantified for total Ca confidently (including bile, ascites fluid, and pancreas), (4) not all tissues were analyzed, such as the brain, bone, lungs, endothelium, or GIT tissue, which are all possible Ca sinks during infection, (5) we analyzed Ca concentrations at one singular timepoint and did not measure Ca flux during 6 h, and (6) the total amount of iCa “absent” from circulation at 6 h after LPS was 23 mg, which is a minuscule quantity (0.01%) compared to the whole-body pool of approximately 226 g Ca in this size pig (Pettey et al., 2015).

### Gastrointestinal physiology

Immune activation can also cause extensive disruptions to GIT homeostasis throughout the alimentary canal (Liang et al., 2005). Maintaining GIT health is obviously important because it (digestion and absorption) is key to livestock performance. Therefore, our second objective was to evaluate some aspects of GIT physiology during immune activation. We hypothesized upper GIT motility would decrease after LPS, characterized by increased digesta weights in the stomach and small intestine (due to LPS-induced GIT ischemia; Fink, 1991), but that large intestine contents would be decreased as a consequence of increased motility and diarrhea.

There was no difference in stomach contents in LPS and CON pigs after 6 h. Prior evidence suggests gastric motility, transit, and emptying are blunted by LPS in rodents (Wirthlin et al., 1996; De Winter et al., 2002). A likely explanation as to why gastric contents were not increased by LPS herein is because four out of six LPS-administered pigs vomited and this digesta was unquantified. If the amount of vomit was monitored and included, the stomach contents would have likely been statistically increased by LPS. Understanding the evolutionary reasons why immune activation appears to reduce gastric emptying may provide clues as to why sick animals become anorexic.

Pigs administered LPS had increased stomach mucosa hemorrhaging, which has been observed previously in rodents (Robinson et al., 2005) and humans (Altemeier et al., 1972). Ischemia of the splanchnic bed is thought to injure the stomach lining by enabling increased free radical damage (Yoshikawa et al., 1989). Gastric bleeding is common in stressed pigs or certain dietary conditions (i.e., small feed particle size; infrequent or off-feed events; Blackshaw and Kelly, 1980; Lawrence et al., 1998). Having a better understanding of how immune activation causes stomach hemorrhaging has practical implications to commercial pig farming.

No treatment differences in small intestine contents on both a DM or wet basis were detected, which suggests small intestine motility was unaffected by LPS. Small intestine smooth muscle activity decreases after intraperitoneal LPS administration or cecal ligation in rats (Overhaus et al., 2004). However, in fasted pigs receiving 24 h of continuously infused i.v. LPS, jejunal motility initially increases (Bruins et al., 2003). Small intestine motility following LPS may be confounded with inappetence and can vary by experiment through effects of route, type, and dose of LPS administration (acute or continuous) and the timing of motility or transit measurements relative to LPS (Cullen et al., 1999). Further, extrapolating measures of small intestinal contents to motility dynamics herein has limitations because differential effects of gastric emptying would ostensibly blunt the ability to detect an increase in small intestine digesta. Presumably due to typically reduced stomach emptying, LPS also decreases bile output, pancreatic secretions (i.e., bicarbonate), and nutrient absorption (Cullen et al., 1999). This could explain the tendency for reduced small intestine pH in LPS-administered pigs. In summary, it is unclear how small intestine motility was influenced by LPS in this experiment, but pH was reduced and this may suggest altered GIT digestive capacity.

Administering LPS increases colonic motility and transit (Spates et al., 1998), and this is corroborated with reduced large intestine contents and increased feces observed herein. Additionally, fecal pH was decreased in LPS relative to CON pigs and this agrees with observations in septic humans (Osuka et al., 2012), indicating reduced absorption and/or increased volatile fatty acid production. Overall, LPS induced emptying of large intestine contents and caused diarrhea, which suggested decreased absorption of fermentation products (Zhao et al., 2023).

The effects of LPS on the GIT are multifaceted. Smooth muscle cells of the GIT contain toll-like receptors that reduce GIT motility once activated (Scirocco et al., 2010). Other inflammatory components presumably influence GIT motility during an LPS response including nitric oxide (Wirthlin et al., 1996), lipid peroxides (De Filippis et al., 2007), and prostaglandins (Rebollar et al., 2002). Immune activation-induced dysmotility and hypophagia are ostensibly evolutionary strategies the animal utilizes to survive an immune challenge. Anorexia decreases nutrient availability for proliferating enteric pathogens (Wang et al., 2016) and increased intestinal motility decreases the enteric pathogen load (Govil and Pal, 2020). In this scenario, although LPS was administered peripherally, the animal still employs GIT strategies in an attempt to minimize the presumed enteric antigens.

## Conclusion

Administering intramuscular LPS elicited acute immune activation and successfully induced hypocalcemia. We were in large part unsuccessful in determining where circulating Ca is partitioned following LPS administration. Our inability to analyze Ca in all tissues or accurately detect small Ca differences in large Ca pools prevented an accurate assessment of Ca trafficking. Additionally, LPS markedly affected multiple aspects of GIT physiology including stark gastric hemorrhaging; changes presumably associated with impaired GIT health. Having a better understanding of the etiology of LPS-induced hypocalcemia and the influence of immune activation on GIT biology would likely provide insight into developing strategies to maximize farm animal productivity during health challenges.

## Supplementary Material

skae073_suppl_Supplementary_Tables_S1-S13

## Abbreviations

AA: amino acid
AST: aspartate aminotransferase
APP: acute phase protein
BUN: blood urea nitrogen
BW: body weight
CON: control
DM: dry matter
GGT: gamma-glutamyl transferase
GIT: gastrointestinal tract
iCa: ionized calcium
LPS: lipopolysaccharide
pCO_2_: partial pressure of carbon dioxide
pO_2_: partial pressure of oxygen
PTH: parathyroid hormone
sO_2_%: oxygen saturation
TCO_2_: total carbon dioxide
