# Supplementary material for: Calcium trafficking and gastrointestinal physiology following an acute lipopolysaccharide challenge in pigs
Source: J Anim Sci. 2024 Mar 14;102:skae073. doi: 10.1093/jas/skae073 (PMC11034434; doi:10.1093/jas/skae073)
Supplement: skae073_suppl_Supplementary_Tables_S1-S13 [file skae073_suppl_supplementary_tables_s1-s13.docx]

| **Supplementary Table 1.** Effects of lipopolysaccharide (LPS) on Na concentrations | | | | |
| --- | --- | --- | --- | --- |
| Location | Treatment | | SEM |  |
|  | CON^1^ | LPS^2^ |  | *P* |
| Tissues, mg/kg DM^3^ |  |  |  |  |
| Adipose | 932 | 832 | 97 | 0.48 |
| Heart | 5443 | 5441 | 192 | 0.99 |
| Kidney | 6177 | 8410 | 521 | 0.01 |
| Liver | 3670 | 4136 | 148 | 0.05 |
| Muscle | 1884 | 1942 | 111 | 0.72 |
| Pancreas | 3281 | 3165 | 259 | 0.76 |
| Spleen | 3777 | 3825 | 240 | 0.89 |
|  |  |  |  |  |
| Digesta, g/kg DM |  |  |  |  |
| Stomach Contents | 9.1 | 28.5 | 7.1 | 0.09 |
| Small Intestine Contents | 27.9 | 26.3 | 3.3 | 0.75 |
| Large Intestine Contents | 9.54 | 9.67 | 1.82 | 0.96 |
| Feces | 3.03 | 5.63 | 1.19 | 0.15 |
|  |  |  |  |  |
| Fluids, mg/L wet |  |  |  |  |
| Ascites | *n.d.*^4^ | *n.d.* | *.* | *.* |
| Bile | *n.d.* | *n.d.* | *.* | *.* |
| Urine | *n.d.* | *n.d.* | *.* | *.* |
| ^1^CON: Pigs administered 2 mL sterile saline.  ^2^LPS: Pigs administered 40 µg/kg BW LPS (*Escherichia coli* O55:B5) in 2 mL saline.  ^3^DM: Dry matter.  ^4^*n.d.:* No data. | | | | |

| **Supplementary Table 2.** Effects of lipopolysaccharide (LPS) on Mg concentrations | | | | |
| --- | --- | --- | --- | --- |
| Location | Treatment | | SEM |  |
|  | CON^1^ | LPS^2^ |  | *P* |
| Tissues, mg/kg DM^3^ |  |  |  |  |
| Adipose | 37.0 | 32.2 | 3.2 | 0.31 |
| Heart | 740 | 714 | 24 | 0.46 |
| Kidney | 715 | 749 | 41 | 0.57 |
| Liver | 727 | 736 | 24 | 0.79 |
| Muscle | 1087 | 1090 | 43 | 0.95 |
| Pancreas | 877 | 891 | 50 | 0.84 |
| Spleen | 985 | 934 | 51 | 0.50 |
|  |  |  |  |  |
| Digesta, mg/kg DM |  |  |  |  |
| Stomach Contents | 222 | 331 | 61 | 0.24 |
| Small Intestine Contents | 986 | 776 | 55 | 0.03 |
| Large Intestine Contents | 6818 | 8505 | 1955 | 0.56 |
| Feces | 8873 | 7426 | 480 | 0.06 |
|  |  |  |  |  |
| Fluids, mg/L wet |  |  |  |  |
| Ascites | 17.0 | 18.2 | 2.1 | 0.69 |
| Bile | 55.3 | 59.3 | 8.6 | 0.75 |
| Urine | 96.0 | 32.6 | 17.2 | 0.03 |
| ^1^CON: Pigs administered 2 mL sterile saline.  ^2^LPS: Pigs administered 40 µg/kg BW LPS (*Escherichia coli* O55:B5) in 2 mL saline.  ^3^DM: Dry matter. | | | | |

| **Supplementary Table 3.** Effects of lipopolysaccharide (LPS) on P concentrations | | | | |
| --- | --- | --- | --- | --- |
| Location | Treatment | | SEM |  |
|  | CON^1^ | LPS^2^ |  | *P* |
| Tissues, mg/kg DM^3^ |  |  |  |  |
| Adipose | 419 | 371 | 24 | 0.19 |
| Heart | 7074 | 6603 | 334 | 0.34 |
| Kidney | 9309 | 9204 | 615 | 0.91 |
| Liver | 11427 | 10743 | 539 | 0.39 |
| Muscle | 8628 | 8266 | 326 | 0.45 |
| Pancreas | 11920 | 11730 | 712 | 0.85 |
| Spleen | 12443 | 11113 | 691 | 0.20 |
|  |  |  |  |  |
| Digesta, g/kg DM |  |  |  |  |
| Stomach Contents | 1.42 | 1.57 | 0.19 | 0.61 |
| Small Intestine Contents | 3.85 | 3.57 | 4.47 | 0.66 |
| Large Intestine Contents | 16.9 | 22.6 | 4.9 | 0.43 |
| Feces | 19.5 | 16.8 | 1.3 | 0.15 |
|  |  |  |  |  |
| Fluids, mg/L we |  |  |  |  |
| Ascites | 109 | 143 | 8 | 0.01 |
| Bile | 524 | 407 | 59 | 0.19 |
| Urine | 488 | 683 | 115 | 0.26 |
| ^1^CON: Pigs administered 2 mL sterile saline.  ^2^LPS: Pigs administered 40 µg/kg BW LPS (*Escherichia coli* O55:B5) in 2 mL saline.  ^3^DM: Dry matter. | | | | |

| **Supplementary Table 4.** Effects of lipopolysaccharide (LPS) on K concentrations | | | | |
| --- | --- | --- | --- | --- |
| Location | Treatment | | SEM |  |
|  | CON^1^ | LPS^2^ |  | *P* |
| Tissues, mg/kg DM^3^ |  |  |  |  |
| Adipose | 511 | 439 | 35 | 0.18 |
| Heart | 11274 | 10928 | 362 | 0.51 |
| Kidney | 10064 | 10548 | 617 | 0.59 |
| Liver | 10834 | 10411 | 308 | 0.35 |
| Muscle | 16563 | 16369 | 596 | 0.82 |
| Pancreas | 12624 | 11235 | 604 | 0.13 |
| Spleen | 18539 | 17750 | 923 | 0.56 |
|  |  |  |  |  |
| Digesta, g/kg DM |  |  |  |  |
| Stomach Contents | 2.32 | 2.92 | 0.60 | 0.50 |
| Small Intestine Contents | 6.58 | 6.88 | 1.09 | 0.85 |
| Large Intestine Contents | 8.20 | 7.91 | 1.42 | 0.89 |
| Feces | 12.0 | 10.6 | 1.1 | 0.41 |
|  |  |  |  |  |
| Fluids, mg/L wet |  |  |  |  |
| Ascites | 319 | 392 | 39 | 0.22 |
| Bile | 371 | 436 | 38 | 0.25 |
| Urine | 2899 | 3019 | 511 | 0.87 |
| ^1^CON: Pigs administered 2 mL sterile saline.  ^2^LPS: Pigs administered 40 µg/kg BW LPS (*Escherichia coli* O55:B5) in 2 mL saline.  ^3^DM: Dry matter. | | | | |

| **Supplementary Table 5.** Effects of lipopolysaccharide (LPS) on Cr concentrations | | | | |
| --- | --- | --- | --- | --- |
| Location | Treatment | | SEM |  |
|  | CON^1^ | LPS^2^ |  | *P* |
| Tissues, µg/kg DM^3^ |  |  |  |  |
| Adipose | 145 | 127 | 17 | 0.45 |
| Heart | 457 | 519 | 86 | 0.62 |
| Kidney | 367 | 394 | 32 | 0.57 |
| Liver | 351 | 346 | 57 | 0.95 |
| Muscle | 399 | 398 | 35 | 0.99 |
| Pancreas | 598 | 546 | 129 | 0.78 |
| Spleen | 627 | 925 | 211 | 0.34 |
|  |  |  |  |  |
| Digesta, mg/kg DM |  |  |  |  |
| Stomach Contents | 1.09 | 1.06 | 0.15 | 0.92 |
| Small Intestine Contents | 1.82 | 1.35 | 2.86 | 0.28 |
| Large Intestine Contents | 15.2 | 20.4 | 4.7 | 0.46 |
| Feces | 19.4 | 16.9 | 1.0 | 0.12 |
|  |  |  |  |  |
| Fluids, µg/L wet |  |  |  |  |
| Ascites | *n.d.*^4^ | *n.d.* | *.* | *.* |
| Bile | *n.d.* | *n.d.* | *.* | *.* |
| Urine | *n.d.* | *n.d.* | *.* | *.* |
| ^1^CON: Pigs administered 2 mL sterile saline.  ^2^LPS: Pigs administered 40 µg/kg BW LPS (*Escherichia coli* O55:B5) in 2 mL saline.  ^3^DM: Dry matter.  ^4^*n.d.:* No data. | | | | |

| **Supplementary Table 6.** Effects of lipopolysaccharide (LPS) on Mn concentrations | | | | |
| --- | --- | --- | --- | --- |
| Location | Treatment | | SEM |  |
|  | CON^1^ | LPS^2^ |  | *P* |
| Tissues, µg/kg DM^3^ |  |  |  |  |
| Adipose | *n.d.*^4^ | *n.d.* | *.* | *.* |
| Heart | *n.d.* | *n.d.* | *.* | *.* |
| Kidney | 5978 | 6264 | 480 | 0.68 |
| Liver | 11254 | 10708 | 497 | 0.46 |
| Muscle | *n.d.* | *n.d.* | *.* | *.* |
| Pancreas | 6040 | 5916 | 572 | 0.88 |
| Spleen | 1143 | 1181 | 76 | 0.73 |
|  |  |  |  |  |
| Digesta, mg/kg DM |  |  |  |  |
| Stomach Contents | 13.9 | 8.4 | 2.0 | 0.09 |
| Small Intestine Contents | 32.5 | 15.3 | 4.2 | 0.02 |
| Large Intestine Contents | 335 | 472 | 112 | 0.41 |
| Feces | 449 | 401 | 12 | 0.02 |
|  |  |  |  |  |
| Fluids, µg/L wet |  |  |  |  |
| Ascites | 10.8 | 15.4 | 2.3 | 0.20 |
| Bile | 772 | 550 | 153 | 0.33 |
| Urine | *n.d.* | *n.d.* | *.* | *.* |
| ^1^CON: Pigs administered 2 mL sterile saline.  ^2^LPS: Pigs administered 40 µg/kg BW LPS (*Escherichia coli* O55:B5) in 2 mL saline.  ^3^DM: Dry matter.  ^4^*n.d.:* No data. | | | | |

| **Supplementary Table 7.** Effects of lipopolysaccharide (LPS) on Fe concentrations | | | | |
| --- | --- | --- | --- | --- |
| Location | Treatment | | SEM |  |
|  | CON^1^ | LPS^2^ |  | *P* |
| Tissues, mg/kg DM |  |  |  |  |
| Adipose | 7.22 | 7.65 | 0.82 | 0.72 |
| Heart | 179 | 163 | 10 | 0.28 |
| Kidney | 193 | 246 | 21 | 0.11 |
| Liver | 502 | 423 | 41 | 0.20 |
| Muscle | 31.6 | 32.1 | 1.7 | 0.85 |
| Pancreas | 65.8 | 64.5 | 6.1 | 0.89 |
| Spleen | 415 | 658 | 49 | <0.01 |
|  |  |  |  |  |
| Digesta, mg/kg DM |  |  |  |  |
| Stomach Contents | 102 | 127 | 17 | 0.34 |
| Small Intestine Contents | 153 | 155 | 14 | 0.92 |
| Large Intestine Contents | 1291 | 1822 | 453 | 0.43 |
| Feces | 1341 | 1437 | 284 | 0.82 |
|  |  |  |  |  |
| Fluids, mg/L wet |  |  |  |  |
| Ascites | 0.78 | 1.33 | 0.21 | 0.09 |
| Bile | 0.92 | 0.58 | 0.22 | 0.30 |
| Urine | *n.d.*^4^ | *n.d.* | *.* | *.* |
| ^1^CON: Pigs administered 2 mL sterile saline.  ^2^LPS: Pigs administered 40 µg/kg BW LPS (*Escherichia coli* O55:B5) in 2 mL saline.  ^3^DM: Dry matter.  ^4^*n.d.:* No data. | | | | |

| **Supplementary Table 8.** Effects of lipopolysaccharide (LPS) on Co concentrations | | | | |
| --- | --- | --- | --- | --- |
| Location | Treatment | | SEM |  |
|  | CON^1^ | LPS^2^ |  | *P* |
| Tissues, µg/kg DM^3^ |  |  |  |  |
| Adipose | *n.d.*^4^ | *n.d.* | *.* | *.* |
| Heart | 18.3 | 22.2 | 2.9 | 0.37 |
| Kidney | 37.2 | 42.9 | 6.2 | 0.52 |
| Liver | 29.0 | 31.3 | 2.9 | 0.58 |
| Muscle | 16.1 | 16.6 | 3.4 | 0.92 |
| Pancreas | *n.d.* | *n.d.* | *.* | *.* |
| Spleen | *n.d.* | *n.d.* | *.* | *.* |
|  |  |  |  |  |
| Digesta, µg/kg DM |  |  |  |  |
| Stomach Contents | *n.d.* | *n.d.* | *.* | *.* |
| Small Intestine Contents | 38.8 | 36.5 | 8.0 | 0.85 |
| Large Intestine Contents | 351 | 537 | 100 | 0.23 |
| Feces | 460 | 418 | 24 | 0.25 |
|  |  |  |  |  |
| Fluids, µg/L wet |  |  |  |  |
| Ascites | *n.d.* | *n.d.* | *.* | *.* |
| Bile | *n.d.* | *n.d.* | *.* | *.* |
| Urine | *n.d.* | *n.d.* | *.* | *.* |
| ^1^CON: Pigs administered 2 mL sterile saline.  ^2^LPS: Pigs administered 40 µg/kg BW LPS (*Escherichia coli* O55:B5) in 2 mL saline.  ^3^DM: Dry matter.  ^4^*n.d.:* No data. | | | | |

| **Supplementary Table 9.** Effects of lipopolysaccharide (LPS) on Cu concentrations | | | | |
| --- | --- | --- | --- | --- |
| Location | Treatment | | SEM |  |
|  | CON^1^ | LPS^2^ |  | *P* |
| Tissues, mg/kg DM^3^ |  |  |  |  |
| Adipose | *n.d.*^4^ | *n.d.* | *.* | *.* |
| Heart | 16.8 | 14.0 | 0.8 | 0.04 |
| Kidney | 32.1 | 36.2 | 3.9 | 0.47 |
| Liver | 23.7 | 23.4 | 1.7 | 0.90 |
| Muscle | *n.d.* | *n.d.* | *.* | *.* |
| Pancreas | 7.88 | 5.96 | 0.97 | 0.20 |
| Spleen |  |  |  |  |
|  |  |  |  |  |
| Digesta, mg/kg DM |  |  |  |  |
| Stomach Contents | *n.d.* | *n.d.* | *.* | *.* |
| Small Intestine Contents | 25.2 | 18.2 | 1.3 | <0.01 |
| Large Intestine Contents | 125 | 174 | 41 | 0.42 |
| Feces | 157 | 144 | 8 | 0.30 |
|  |  |  |  |  |
| Fluids, mg/L wet | *n.d.* | *n.d.* | *.* | *.* |
| Ascites | 0.43 | 1.13 | 0.06 | <0.01 |
| Bile | 1.98 | 1.23 | 0.26 | 0.06 |
| Urine | 0.20 | 0.12 | 0.03 | 0.10 |
| ^1^CON: Pigs administered 2 mL sterile saline.  ^2^LPS: Pigs administered 40 µg/kg BW LPS (*Escherichia coli* O55:B5) in 2 mL saline.  ^3^DM: Dry matter.  ^4^*n.d.:* No data. | | | | |

| **Supplementary Table 10.** Effects of lipopolysaccharide (LPS) on Zn concentrations | | | | |
| --- | --- | --- | --- | --- |
| Location | Treatment | | SEM |  |
|  | CON^1^ | LPS^2^ |  | *P* |
| Tissues, mg/kg DM^3^ |  |  |  |  |
| Adipose | 2.93 | 2.42 | 0.21 | 0.12 |
| Heart | 66.0 | 67.6 | 5.1 | 0.83 |
| Kidney | 92.2 | 104.2 | 5.6 | 0.16 |
| Liver | 310 | 380 | 45 | 0.30 |
| Muscle | 36.7 | 35.9 | 1.7 | 0.75 |
| Pancreas | 253 | 296 | 33 | 0.38 |
| Spleen | 84.8 | 80.7 | 3.7 | 0.45 |
|  |  |  |  |  |
| Digesta, mg/kg DM |  |  |  |  |
| Stomach Contents | 44.9 | 35.6 | 8.3 | 0.45 |
| Small Intestine Contents | 88.1 | 51.9 | 11.0 | 0.04 |
| Large Intestine Contents | 947 | 1291 | 311 | 0.45 |
| Feces | 1344 | 1176 | 41 | 0.02 |
|  |  |  |  |  |
| Fluids, mg/L wet |  |  |  |  |
| Ascites | 0.18 | 0.45 | 0.04 | <0.01 |
| Bile | 0.18 | 0.23 | 0.06 | 0.54 |
| Urine | 0.26 | 0.73 | 0.10 | <0.01 |
| ^1^CON: Pigs administered 2 mL sterile saline.  ^2^LPS: Pigs administered 40 µg/kg BW LPS (*Escherichia coli* O55:B5) in 2 mL saline.  ^3^DM: Dry matter. | | | | |

| **Supplementary Table 11.** Effects of lipopolysaccharide (LPS) on Se concentrations | | | | |
| --- | --- | --- | --- | --- |
| Location | Treatment | | SEM |  |
|  | CON^1^ | LPS^2^ |  | *P* |
| Tissues, µg/kg DM^3^ |  |  |  |  |
| Adipose | 69.4 | 66.1 | 10.8 | 0.83 |
| Heart | 1696 | 1434 | 162 | 0.28 |
| Kidney | 7060 | 6110 | 362 | 0.09 |
| Liver | 2708 | 2647 | 127 | 0.74 |
| Muscle | 1285 | 1483 | 73 | 0.08 |
| Pancreas | 2008 | 2080 | 123 | 0.69 |
| Spleen | 1759 | 1603 | 124 | 0.39 |
|  |  |  |  |  |
| Digesta, µg/kg DM |  |  |  |  |
| Stomach Contents | 350 | 417 | 66 | 0.49 |
| Small Intestine Contents | 669 | 850 | 78 | 0.14 |
| Large Intestine Contents | 1172 | 1576 | 316 | 0.39 |
| Feces | 1530 | 1498 | 90 | 0.80 |
|  |  |  |  |  |
| Fluids, µg/L wet |  |  |  |  |
| Ascites | 41.8 | 127.3 | 4.3 | <0.01 |
| Bile | 39.1 | 37.4 | 5.0 | 0.82 |
| Urine | 428 | 357 | 61 | 0.44 |
| ^1^CON: Pigs administered 2 mL sterile saline.  ^2^LPS: Pigs administered 40 µg/kg BW LPS (*Escherichia coli* O55:B5) in 2 mL saline.  ^3^DM: Dry matter. | | | | |

| **Supplementary Table 12.** Effects of lipopolysaccharide (LPS) on Mo concentrations | | | | |
| --- | --- | --- | --- | --- |
| Location | Treatment | | SEM |  |
|  | CON^1^ | LPS^2^ |  | *P* |
| Tissues, µg/kg DM^3^ |  |  |  |  |
| Adipose | *n.d.*^4^ | *n.d.* | *.* | *.* |
| Heart | 248 | 161 | 27 | 0.05 |
| Kidney | 2740 | 2642 | 143 | 0.64 |
| Liver | 3126 | 2927 | 277 | 0.62 |
| Muscle | *n.d.* | *n.d.* | *.* | *.* |
| Pancreas | 453 | 511 | 39 | 0.32 |
| Spleen | 114 | 146 | 13 | 0.12 |
|  |  |  |  |  |
| Digesta, µg/kg DM |  |  |  |  |
| Stomach Contents | 390 | 332 | 47 | 0.41 |
| Small Intestine Contents | 331 | 255 | 21 | 0.03 |
| Large Intestine Contents | 1088 | 1188 | 251 | 0.79 |
| Feces | 1142 | 1202 | 204 | 0.84 |
|  |  |  |  |  |
| Fluids, µg/L wet |  |  |  |  |
| Ascites | 12.7 | 10.6 | 1.4 | 0.33 |
| Bile | 9.18 | 11.17 | 1.47 | 0.36 |
| Urine | 580 | 545 | 100 | 0.81 |
| ^1^CON: Pigs administered 2 mL sterile saline.  ^2^LPS: Pigs administered 40 µg/kg BW LPS (*Escherichia coli* O55:B5) in 2 mL saline.  ^3^DM: Dry matter.  ^4^*n.d.:* No data. | | | | |

| **Supplementary Table 13.** Effects of lipopolysaccharide (LPS) on Cd concentrations | | | | |
| --- | --- | --- | --- | --- |
| Location | Treatment | | SEM |  |
|  | CON^1^ | LPS^2^ |  | *P* |
| Tissues, µg/kg DM^3^ |  |  |  |  |
| Adipose | *n.d.*^4^ | *n.d.* | *.* | *.* |
| Heart | *n.d.* | *n.d.* | *.* | *.* |
| Kidney | 190 | 233 | 30 | 0.34 |
| Liver | 39.4 | 36.9 | 2.5 | 0.49 |
| Muscle | 16.1 | 18.4 | 3.2 | 0.63 |
| Pancreas | 13.9 | 14.6 | 1.7 | 0.80 |
| Spleen | *n.d.* | *n.d.* | *.* | *.* |
|  |  |  |  |  |
| Digesta, µg/kg DM |  |  |  |  |
| Stomach Contents | 20.0 | 38.8 | 7.1 | 0.10 |
| Small Intestine Contents | 92.5 | 75.2 | 10.7 | 0.28 |
| Large Intestine Contents | 142 | 243 | 35 | 0.07 |
| Feces | 190 | 165 | 3 | <0.01 |
|  |  |  |  |  |
| Fluids, µg/L wet |  |  |  |  |
| Ascites | *n.d.* | *n.d.* | *.* | *.* |
| Bile | *n.d.* | *n.d.* | *.* | *.* |
| Urine | *n.d.* | *n.d.* | *.* | *.* |
| ^1^CON: Pigs administered 2 mL sterile saline.  ^2^LPS: Pigs administered 40 µg/kg BW LPS (*Escherichia coli* O55:B5) in 2 mL saline.  ^3^DM: Dry matter.  ^4^*n.d.:* No data. | | | | |
